# Optimizing Filter-Probe Diffusion Weighting in the Rat Spinal Cord for Human Translation

**DOI:** 10.3389/fnins.2017.00706

**Published:** 2017-12-19

**Authors:** Matthew D. Budde, Nathan P. Skinner, L. Tugan Muftuler, Brian D. Schmit, Shekar N. Kurpad

**Affiliations:** ^1^Department of Neurosurgery, Medical College of Wisconsin, Milwaukee, WI, United States; ^2^Medical Scientist Training Program, Medical College of Wisconsin, Milwaukee, WI, United States; ^3^Department of Biomedical Engineering, Marquette University and the Medical College of Wisconsin, Milwaukee, WI, United States

**Keywords:** diffusion tensor imaging, double diffusion encoding, spinal cord injury, magnetic resonance imaging

## Abstract

Diffusion tensor imaging (DTI) is a promising biomarker of spinal cord injury (SCI). In the acute aftermath, DTI in SCI animal models consistently demonstrates high sensitivity and prognostic performance, yet translation of DTI to acute human SCI has been limited. In addition to technical challenges, interpretation of the resulting metrics is ambiguous, with contributions in the acute setting from both axonal injury and edema. Novel diffusion MRI acquisition strategies such as double diffusion encoding (DDE) have recently enabled detection of features not available with DTI or similar methods. In this work, we perform a systematic optimization of DDE using simulations and an *in vivo* rat model of SCI and subsequently implement the protocol to the healthy human spinal cord. First, two complementary DDE approaches were evaluated using an orientationally invariant or a filter-probe diffusion encoding approach. While the two methods were similar in their ability to detect acute SCI, the filter-probe DDE approach had greater predictive power for functional outcomes. Next, the filter-probe DDE was compared to an analogous single diffusion encoding (SDE) approach, with the results indicating that in the spinal cord, SDE provides similar contrast with improved signal to noise. In the SCI rat model, the filter-probe SDE scheme was coupled with a reduced field of view (rFOV) excitation, and the results demonstrate high quality maps of the spinal cord without contamination from edema and cerebrospinal fluid, thereby providing high sensitivity to injury severity. The optimized protocol was demonstrated in the healthy human spinal cord using the commercially-available diffusion MRI sequence with modifications only to the diffusion encoding directions. Maps of axial diffusivity devoid of CSF partial volume effects were obtained in a clinically feasible imaging time with a straightforward analysis and variability comparable to axial diffusivity derived from DTI. Overall, the results and optimizations describe a protocol that mitigates several difficulties with DTI of the spinal cord. Detection of acute axonal damage in the injured or diseased spinal cord will benefit the optimized filter-probe diffusion MRI protocol outlined here.

## Introduction

A noninvasive biomarker of spinal cord injury (SCI) severity is a highly-sought goal that could aid clinical decision-making and facilitate clinical trial enrollment and outcomes. Diffusion weighted imaging (DWI) is a magnetic resonance imaging (MRI) technique uniquely sensitive to microscopic injury and has shown promise as a biomarker of SCI. However, although preclinical studies have consistently shown diffusion tensor imaging (DTI) captures microscopic injury not evident through other MRI contrasts (Krzyzak et al., 2005; Deo et al., 2006; Gaviria et al., 2006; Loy et al., 2007; Herrera et al., 2008; Shemesh and Cohen, 2008; Kim et al., 2010; Sundberg et al., 2010; Tu et al., 2010; Mondragon-Lozano et al., 2013; Kelley et al., 2014; Wang et al., 2014; Li et al., 2015; Patel et al., 2016; Skinner et al., 2016), the adoption of DTI to human SCI, and acute SCI in particular, has been limited to only a small number of studies (Cheran et al., 2011; Endo et al., 2011; Vedantam et al., 2015; Shanmuganathan et al., 2017). The lack of translation can be attributed to many factors, but technical challenges of imaging the spinal cord along with difficulties in interpretation are prominent hurdles to clinical feasibility and utility for DWI as well as other advanced MRI techniques (Stroman et al., 2014; Wheeler-Kingshott et al., 2014; Martin et al., 2016). Double diffusion encoding has recently been shown to be a rapid and accurate method of assessing the severity of injury in a rat model (Skinner et al., 2016). The goal of this study was to optimize the filter-probe double diffusion encoding for human translation.

Animal studies of experimental SCI convincingly demonstrate microscopic disruption to white matter tracts as detected with DTI both at the lesion site and in regions remote from the site of injury (Krzyzak et al., 2005; Ellingson et al., 2008, 2010; Sundberg et al., 2010; Jirjis et al., 2013). This is often observed as a decrease in the DTI parameter of fractional anisotropy (FA) that is attributed to white matter tract injury, although FA has poor specificity since it reflects a complex combination of multiple pathologies. A reduction in diffusion measured parallel to the fibers, or axial diffusivity (AD), has been shown to be more closely associated with axonal injury. In the acute aftermath of SCI, decreased AD is argued to be caused by the formation of axonal varicosities, or beading, that are observed in experimental SCI (Williams et al., 2014). In a recent human SCI study at 24 h post-injury, AD had a strong relationship with outcome 1 year later (Shanmuganathan et al., 2017). However, the sensitivity of AD to injury can change as the injury-to-imaging interval increases and edema evolves in the initial days to weeks after injury (Leypold et al., 2008). Thus, despite promising results as a biomarker, its use remains limited.

Improvements in the ability of DWI to capture microstructure have been introduced through advanced diffusion encoding techniques, including double diffusion encoding (DDE). DDE, as the term implies, uses two separate diffusion gradient pairs to provide contrast (Cory et al., 1990; Mitra, 1995) unattainable with the traditional Stejskal-Tanner single diffusion encoding (SDE). For a review of the history of DDE with derived measures and accepted nomenclature, see (Shemesh et al., 2016). Most related to neurological injury is the ability of DDE to estimate compartment shape anisotropy or eccentricity. In a previous simulation study (Skinner et al., 2015), microscopic anisotropy estimated from DDE was highly sensitive to axonal injury. Importantly, one DDE variant, referred to as 5-design DDE (Jespersen et al., 2013), has the benefit of being insensitive to the underlying fiber distribution and is termed orientational invariance, which is a particular confound in DTI. Thus, DDE measures of microscopic anisotropy may have a benefit in detecting acute injury to the brain and spinal cord. While DDE has been reported for materials, cells, and tissues, its application to neurological injury has been limited (Shemesh et al., 2014) although applications in aging (Lawrenz et al., 2016) have demonstrated its utility and advantages over more conventional DTI.

Another DDE variant, referred to filter-probe DDE (FP-DDE), was similarly sensitive to axonal beading (Skinner et al., 2015) in a simulation study. It was developed specifically for tissues in which the fiber organization is known a priori and largely coherent, which makes it suitable for the spinal cord but could also be a confound with improper alignment or complex fiber arrangements. In this approach, an initial diffusion “filter” perpendicular to the cord suppresses mobile spins, and a second diffusion “probe” parallel to the cord measures the diffusivity of the unsuppressed spins. It demonstrates a high sensitivity to acute SCI in a rat model and outperforms DTI in stratifying the degree of injury (Skinner et al., 2016). When coupled with a single-voxel diffusion spectroscopy localization, FP-DDE has substantially less post-processing demands than DTI, which is believed to improve its translation to clinical settings. However, FP-DDE is constrained by the requirement that the diffusion encoding directions are aligned with the spinal cord axonal fibers, and curvature of the spinal cord or user error may limit its applicability.

While our previous spectroscopic FP-DDE approach has methodological benefits over an imaging readout, the lack of spatial information limits the visualization of regional injuries that may have important benefits for outcomes along different functional domains such as motor or sensory systems (Martin et al., 2016). Another obvious challenge is that DDE pulse sequences are not readily available on most MRI systems. The inclusion of two separate diffusion encoding gradient pairs increases echo time, resulting in lower signal to noise compared to a SDE. Thus, DDE has other practical considerations that could potentially limit its adoption.

To improve the feasibility and translational potential of DDE for clinical settings, this study systematically compared several different experimental conditions with the goal of developing a protocol capable of clinically-feasible human translation. First, simulations tested the relationship of derived DWI measures with the underlying model of injury with an emphasis on reliability and effects of signal to noise ratio (SNR). Next, the *in vivo* application of alternative DDE methods was examined in a rat model of SCI to identify if orientation-invariant metrics performed as well as metrics aligned with the cord axis. Further, it was assessed whether the contrast obtained from the DDE approach could be captured with a SDE in the spinal cord. Finally, the set of optimized parameters was applied to the healthy human cervical spinal cord to assess reliability of the obtained measures. Collectively, the results define a set of experimental parameters and considerations to provide robust measurements of spinal cord integrity in future studies of patients with spinal cord disease and injury.

## Methods

### Simulations

**Figure 1:**
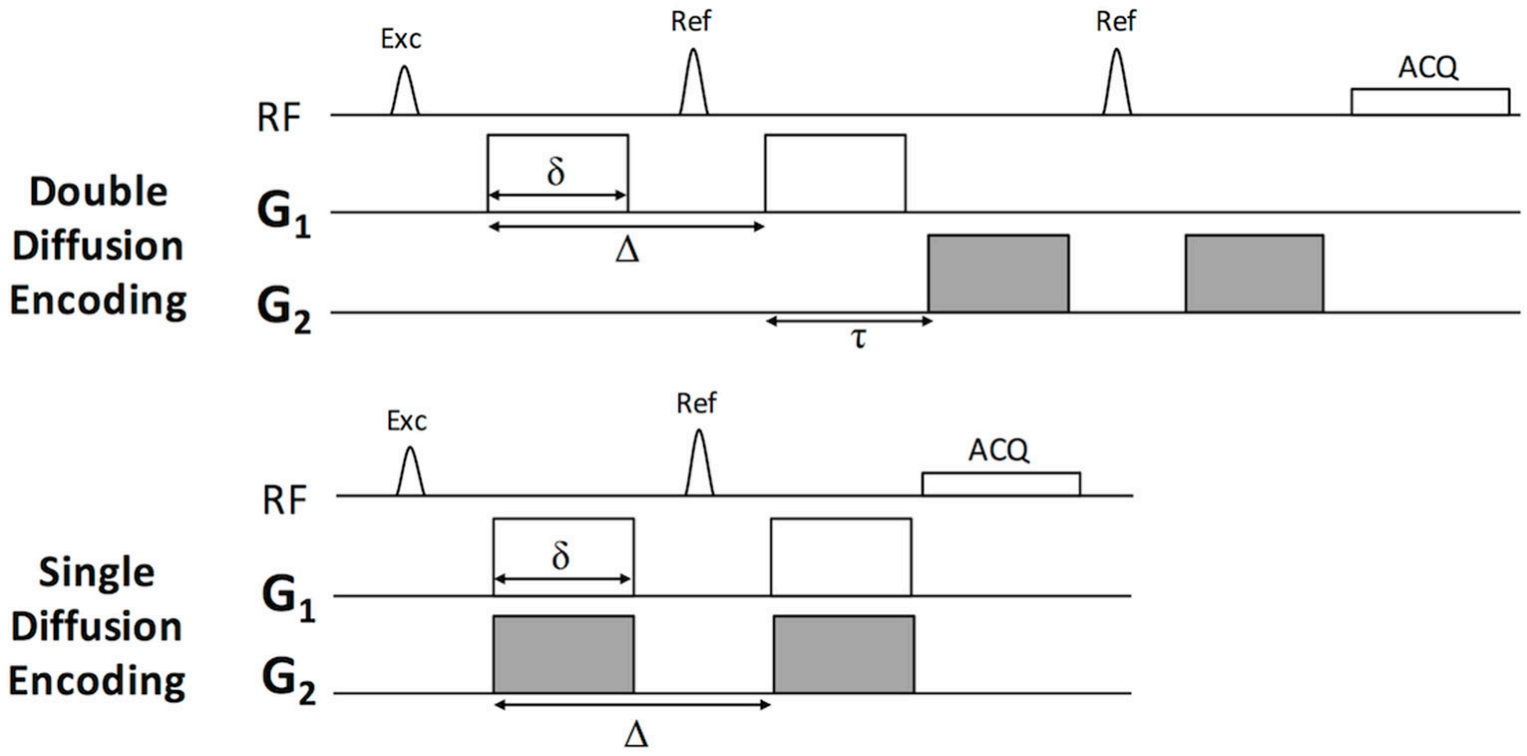
Pulse sequence for Double and Single Diffusion Encoding Techniques. The double diffusion encoding (DDE) sequence consists of two pairs of Stejskal-Tanner diffusion weighting gradients (G_1_ and G_2_) independent in their orientations, timing, and amplitude. The single diffusion encoding (SDE) consists of a single gradient pair. In this work, G_1_ and G_2_ were either parallel or perpendicular to one another and modulated relative to the laboratory frame of reference according to Table 1.

Monte-Carlo simulations were performed to examine the response of diffusion metrics to models of microscopic injury and effects of signal to noise (SNR) using camino diffusion toolkit (Hall and Alexander, 2009) and models of healthy and injured axons as straight and beaded cylinders, respectively, as previously described (Budde and Frank, 2010; Skinner et al., 2015). A range of injured axon fractions and intracellular volume fractions were simulated, and the pulse sequences were those described in Figure 1, Table 1. The simulations each used 50,000 individual spins, 2,000 time-steps, and an intrinsic diffusivity of 1.8 um^2^/ms consistent with the measured longitudinal diffusivity of healthy spinal cord white matter (Budde et al., 2007). To examine the effects of SNR, additional Gaussian noise was added to the phase-sensitive simulated signals to achieve a nominal SNR for the *b* = 0 signal, noting this results in a characteristic Rician noise profile when converted to magnitude signal. The gradient directions are specified in Table 1 and are consistent with the previously reported conditions for the 5-design DDE (Jespersen et al., 2013). The filter-probe DDE was simulated with the gradients consecutive in time as originally proposed (Skinner et al., 2015) in which a diffusion “filter” is applied perpendicular to the known fiber axis and the diffusion “probe” samples the diffusion along the axis in a series of varying gradient amplitudes. The effects of combining the two gradients into a SDE overlapped in time was evaluated (Figure 1 bottom) with other conditions identical.

**Table 1:** Double diffusion encoding schemes and parameters.

	**Orientationally Invariant^a^**	**Filter-Probe^b^**
G_1_ directions	Orthogonal: 60 (12 unique) Parallel: 12	Perpendicular to Cord: 2 (+ & −)
G_1_ *b*-values (s/mm^2^)	2,000	2,000
G_2_ directions	Orthogonal to G_1_: 60 Parallel to G_1_: 12	Parallel to Cord: 2 (+ & −)
G_2_ *b*-values (s/mm^2^)	2,000	9 increments: 0–2,000
Non-DWI (b = 0)	0	0
Total Acquisitions	72	36

a *Jespersen et al. (2013)*.

b *Skinner et al. (2016)*.

The parameters investigated were limited to those that showed nearly perfect specificity for axonal injury in the presence of varying volume fractions in a previous simulation study (Skinner et al., 2015). A measure of microscopic anisotropy, eccentricity (ε), was derived from the 5-design DDE having the feature of orientation invariance defined by

(1) $$\begin{array}{l} \varepsilon =\frac{ln\left(S_{Parallel}\right)-ln\left(S_{Orthogonal}\right)}{q^{4}} \end{array}$$

where S_*Parallel*_ and S_*Orthogonal*_ are the mean signal from the parallel and orthogonal diffusion encoding directions, respectively, and *q* is the diffusion wave vector given by $q=\frac{1}{2\pi }\gamma \text{G}\delta$ with ***G*** and δ the amplitude and duration of the diffusion encoding gradient, respectively. Notably, ε has a strong dependence on the diffusion encoding gradients and may be converted to the parameter microscopic fractional anisotropy to reduce its dependence on experimental conditions (Jespersen et al., 2013). However, the normalization factor includes mean diffusivity (MD) which introduces a minor dependence on intracellular volume fraction (Skinner et al., 2015). For the purposes of this study, ε was obtained under identical experimental conditions and was therefore sufficient to relate to the other derived metrics under the same conditions.

**Figure 2:**
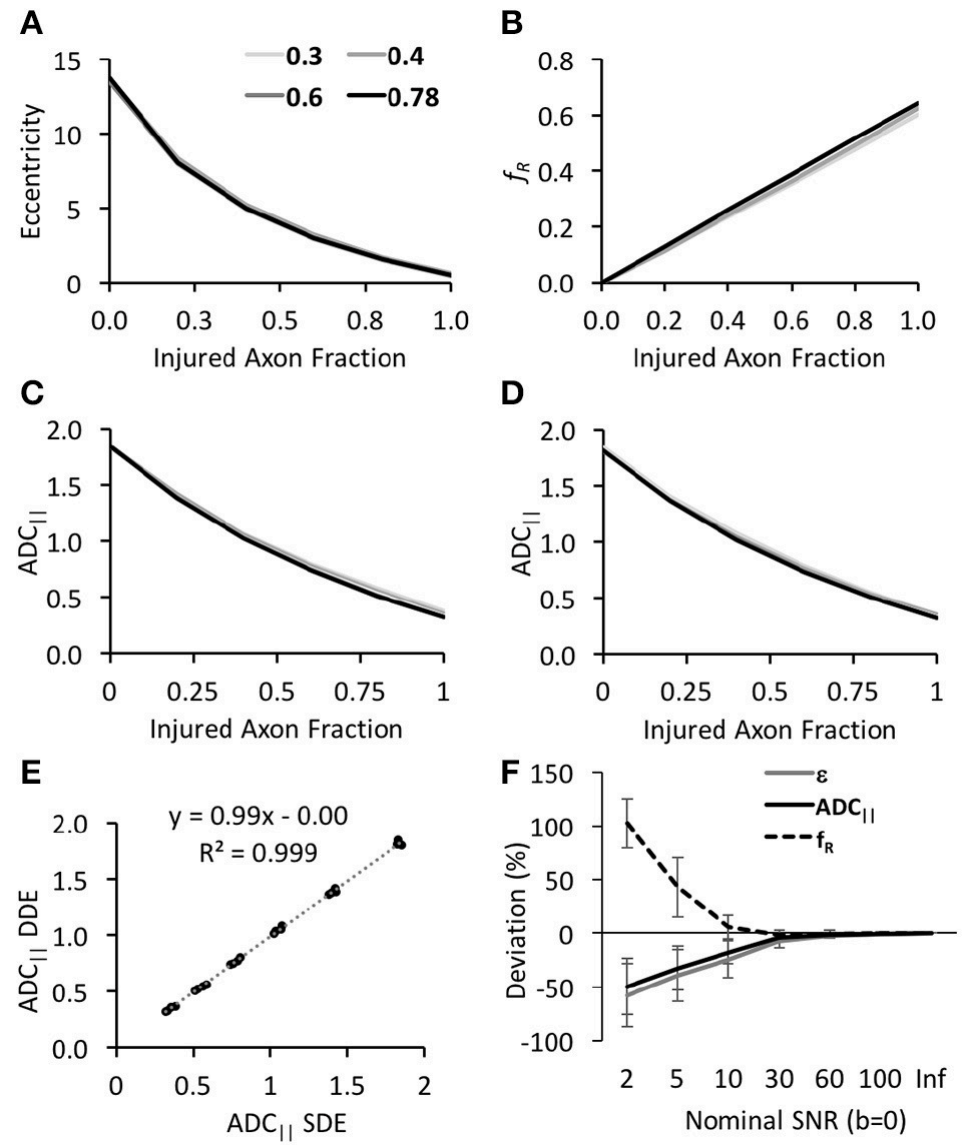
Simulation of DWI schemes and derived parameters. All estimated DWI parameters, including eccentricity **(A)**, restricted fraction **(B)**, and ADC_||_ from the DDE **(C)** or SDE **(D)**, exhibited high sensitivity to the injured axon fraction (x-axis) with minimal differences between different intra-axonal volume fractions (shown as individual lines). Notably, ADC_||_ was nearly identical between the DDE and SDE **(E)** for uniformly-oriented fibers. The estimated parameters showed differential reliability under low SNR with ADC_||_ being most robust to noise **(F)**.

In the filter-probe diffusion encoding scheme (Skinner et al., 2016) a measure of apparent diffusion coefficient along the spinal cord axis (ADC_||_) was obtained in the presence of a perpendicular diffusion filter under the assumption of coherent and uniformly aligned fibers by

(2) $$\begin{array}{l} S_{i}=S_{0}\cdot exp\left(-b\cdot D\right) \end{array}$$

where *S*_*0*_ is the signal measured with without diffusion weighting. Notably, in this specific condition it reflects the signal in the presence of the diffusion “filter” but no parallel diffusion weighting. S_*i*_ reflects the measured signal at each of the *b*-values measured parallel to the fiber axis with *b*=*q*^2^($\Delta -\frac{\delta }{3}$). Likewise, a biexponential model under the same conditions and measurements is given by

(3) $$\begin{array}{l} S_{i}=S_{0}\cdot f_{R}\cdot exp\left(-b\cdot D_{R}\right)+S_{0}\left(1-f_{R}\right)\cdot exp\left(-b\cdot D_{fast}\right) \end{array}$$

where *D*_*R*_ and *D*_*fast*_ reflect the diffusivities of the restricted and fast compartments, respectively, and f_*R*_ reflects the fraction of the restricted signal. All analyses were performed in Matlab using least-squares regression for Equation 2 and the non-linear curve fitting toolbox for Equation 3. The effect of SNR on the estimated parameters (Figure 2) was quantified as the relative error (%) measured as the difference between the estimated and true values (infinite SNR) normalized by the maximum across all simulated axonal and volume fractions.

### Animals

All animal procedures were approved by the Institutional Animal Care and Use Committees (IACUC) at the Medical College of Wisconsin, the Clement J Zablocki VA Medical Center and Northwestern University. A total of 38 female Sprague–Dawley rats (200–250 g) were used. Contusion SCI was performed in rats at the T10 vertebral level of the spinal cord with varying levels of injury severity or sham injury as previously described (Skinner et al., 2016). Briefly, animals were anesthetized and a dorsal laminectomy was performed followed by a weight-drop to deliver a mild, moderate, or severe injury. Sham animals were identical including laminectomy but the weight was not dropped. Naïve animals were used for protocol developments and testing where indicated. Rats underwent locomotor functional assessments using the BBB scale (Basso et al., 1995) at 1 and 30 days post-injury and were scored by a blinded reviewer.

### Rat magnetic resonance imaging

*In vivo* MRI was collected 48 h following the injury procedure using a Bruker 9.4T Biospec System with Paravision (6.0.1). A quadrature volume coil was used for transmission and 4-channel surface coil array for reception centered over the lesion epicenter at the T10 thoracic vertebrae. Animal respiration and temperature were monitored for the duration of the imaging.

**Figure 3:**
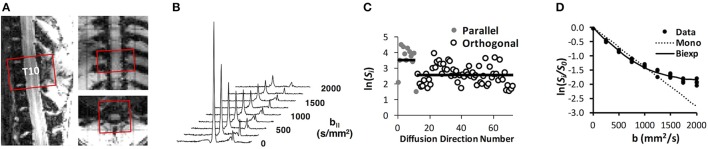
DDE-PRESS Acquisition and Quantification. A voxel was positioned over the T10 vertebral segment **(A)** and aligned with the cord axis, shown here for a sham-injured animal. The integrated signal quantified as the area under the water peak **(B)** was obtained over the range ±2 ppm for subsequent analysis. For orientationally invariant DDE **(C)**, the mean signal from parallel and orthogonal directions were compared to derive eccentricity, whereas for the filter-probe DDE, the diffusivity and restricted fraction **(D)** were derived from a mono- and bi-exponential fit **(D)** to the signals.

The DDE pulse sequence consisted of two separate Stejskal-Tanner diffusion encoding gradients (Figure 1), each with independent direction and amplitude (Table 1), while the diffusion separation and durations were identical (Table 2). To examine the effects of different DDE approaches, a single voxel Point RESolved Spectroscopy (PRESS) acquisition (Bottomley, 1987) was used as previously described (Skinner et al., 2016), with the voxel (10 × 10 × 6 mm^3^) placed at the lesion epicenter and aligned with the spinal cord main axis (Figure 3). The acquisition was cardiac- and respiratory-gated, and other relevant acquisition parameters included sweep width = 4,960 Hz, and number of points = 256. As shown in Table 1, the diffusion parameters for the DDE sequence comparison used identical parameters to the extent possible, including a maximum b-value for each diffusion encoding pair of 2000 s/mm^2^.

**Table 2:** Diffusion weighted experimental parameters.

**Condition**	**1**	**2**	**3**	**4**	**5**
Application	Rat/Simulation	Rat/Simulation	Rat	Rat	Human
Readout	PRESS	PRESS	EPI	EPI	EPI
EPI Segments	–	–	4	4	1
rFOV	–	–	–	2DRF	2DRF
TR (ms)	3,000	3,000	1,500	1,500	2,000
TE (ms)	41	41	61	35	86
FOV (mm^2^)	10	10	25.6 × 25.6	13.5 × 9.6	200
Voxel/Slice thickness (mm)	6	6	1.5	1.5	5
In-plane resolution	–	–	266 × 400μm^2^	150μm^2^	1.43 mm^2^
DWI Scheme^a^	5-design DDE	DDE Filter-Probe	DDE Filter-Probe	SDE Filter-Probe	SDE Filter-Probe
DWI Acquisitions	72	36	36	36	36
Max total *b*-value (s/mm^2^)	4,000	4,000	4,000	3,000	3,000
Diffusion separation Δ (ms)	12	12	12	12	32.5
Diffusion duration (ms)	6	6	6	6	25.4
Mixing Time (τ; ms)	6	6	6	–	–
NEX	1	1	2	2	6
Acquisition Time	3:48	2:09	5:36	5:36	5:26
Outcome Measure(s)	Eccentricity (μm^4^)	ADC_||_ (μm^2^/ms) *f_*R*_* (fraction)	ADC_||_ (μm^2^/ms)	ADC_||_(μm^2^/ms)	ADC_||_ (μm^2^/ms)

a *From Table 1*.

In the naïve spinal cord at T10, the DDE sequence was compared to the SDE using identical diffusion gradients. The SDE enabled a reduction in TE from 61 to 35 ms using a 4-shot echo planar imaging (EPI) readout. Both positive and negative diffusion gradient encoding directions on each axis were acquired to mitigate any directional or cross-term dependence (Neeman et al., 1991).

A reduced field of view (rFOV) excitation scheme (Saritas et al., 2008) was implemented using a 2D excitation (2DRF) echo planar gradient trajectory. The excitation consisted of 16 Gaussian sub-pulses (time-bandwidth product of 2.74) each with 0.2 ms duration and amplitudes modulated by a Gaussian window. The total pulse duration was 6.85 ms. The sequence was evaluated in naïve animals using the DWI-EPI sequence (13.5 × 9.6 mm^2^, 90 × 64 matrix), and compared to rFOV of the same resolution (13.5 × 13.5 mm^2^, 90 × 90 matrix) using outer volume suppression with four 10 mm saturation bands surrounding the acquisition FOV and a separate spectral-selective fat suppression pulse. The 2DRF rFOV did not include any spatial or fat suppression. DWI consisted of 12 directions, b-value of 800 s/mm^2^, with TEs of 24 and 29 ms for the OVS and rFOV, respectively, and identical acquisition times of 3:28 min. Both schemes had 150 μm^2^ in-plane resolution and 2 mm slice thickness. The 2DRF excitation rFOV imaging was combined with the FP-SDE diffusion encoding scheme and applied to the rat SCI at 48 h and 30 days post-injury and performed at the T10 lesion epicenter.

### Human magnetic resonance imaging

All procedures were approved by the Institutional Review Board (IRB) at the Medical College of Wisconsin, and written consent was obtained from all subjects. Three subjects (age range: 28–38 years; 2 male) underwent cervical spine imaging on a 3T General Electric Discovery system using a 12 channel head, neck, and spine receive array. The DWI-EPI vendor-supplied sequence was a single-shot EPI with a 2DRF excitation rFOV. The diffusion gradient directions were modified to include the SDE filter-probe values as shown in Table 1 along with a conventional DTI acquisition with 2 *b*-values of 1,000 and 2,000 s/mm^2^ along 15 directions. Other sequence parameters are listed in Table 2.

### Data analysis

In both naïve and injured animals, maps of parallel diffusivity (ADC_||_) along the spinal cord were calculated according to equation 2 and 3. Notably, *S*_*0*_ reflects the absence of diffusion weighted parallel to the cord but in the presence of the perpendicular diffusion filter. Parameter maps were evaluated using whole-cord region of interest (ROI) analysis. DTI fitting was performed for comparison parameter map quality in full-FOV and rFOV DWI using FSL (Jenkinson et al., 2012). Quantification of ADC_||_ in injured animals used whole-cord averages from a single slice at the T10 injury epicenter. The magnitude spectroscopic signals were integrated between ±2 ppm of the water peak. For the filter-probe DDE, the signals were fit to a monoexponential model. Spinal cord images were analyzed with regions of interest (ROI) using an approach to avoid bias in manual segmentation. DWI_⊥_ images were converted to SNR maps by dividing the images by the standard deviation measured from a region of pure noise. A second ROI surrounding the whole cord was placed in the slice at the lesion epicenter, and only voxels with SNR values above 12 were included in the final mask, which was subsequently transferred to the ADC_||_ map. The whole-cord mean voxel ADC_||_ values were obtained.

The correlations between the resulting metrics and the compression distance (mm) at the moment of injury were assessed using a Pearson's product moment correlation. Likewise, correlations between the diffusion metrics and locomotor function (BBB score) at 30 days post-injury were evaluated with a Pearson correlation. A direct comparison of correlation coefficients between the two different diffusion methods and their relationship with outcome was carried out using the method described in Steiger (1980).

## Results

### Simulations of DWI scheme and SNR dependence

As expected, the three parameters derived from the simulations, ADC_||_, *f*_*R*_, and ε were highly specific to axonal beading even with variations in the intracellular volume fraction (Figure 2). Increasing beading fraction was associated with decreased ε and ADC_||_ and increased *f*_*R*_. While *f*_*R*_ exhibited the most linear relationship with beading fraction (Figure 2B), its slope of 0.64 indicated it did not directly correspond to the true injured fraction. In the filter-probe scheme, ADC_||_ obtained from the DDE or the SDE were identical to one another (*R*^2^ = 0.999; Figures 2C,D), demonstrating the SDE scheme with orthogonal gradients aligned with the fiber axis is equally effective as the DDE under the same conditions. The parameters differed considerably in their accuracy under varying SNR conditions. The nominal SNR values of 10, 30, 60, and 100 for the non-diffusion weighted signals (*b* = 0 s/mm^2^) equated to mean SNR values of 2.6, 7.8, 15.6, and 30.0, respectively, for the diffusion weighted signals across all directions at the highest *b*-values (*b* = 4,000 s/mm^2^). Across all beading and volume fractions and the lowest SNR condition, ADC_||_ exhibited a decrease of 27.3% compared the highest SNR condition whereas eccentricity decreased by 117% and *f*_*R*_ increased by 256%. Furthermore, ADC_||_ was within 10% of its true value at a DW SNR of 11.1%, eccentricity and *f*_*R*_ were within 10% of their true values at SNR values of 34 and 36, respectively, further demonstrating these parameters require greater SNR for accuracy than ADC_||_. It should be noted that these are relative differences and not absolute cutoffs since other factors affect the results under experimental conditions.

### *In vivo* effects of DWI scheme in SCI

The DDE-PRESS was applied to a rat model of spinal cord contusion injury (Figure 3) using parameters similar to those of the simulations (Table 2 condition 1 and 2). The mean SNR across diffusion-weighted spectra at *b* = 4,000 s/mm^2^ were 73.8 (±47.8) and 70.4 (±28.8) for the 5-design DDE and the filter-probe DDE, respectively. Across all animals with varying injury severities (*n* = 17), the average water peak linewidth (FWHM) was 36.8 (±9.3) Hz, and the average coefficient of variation across repeats was 9.8 (±8.3)%.

**Figure 4:**
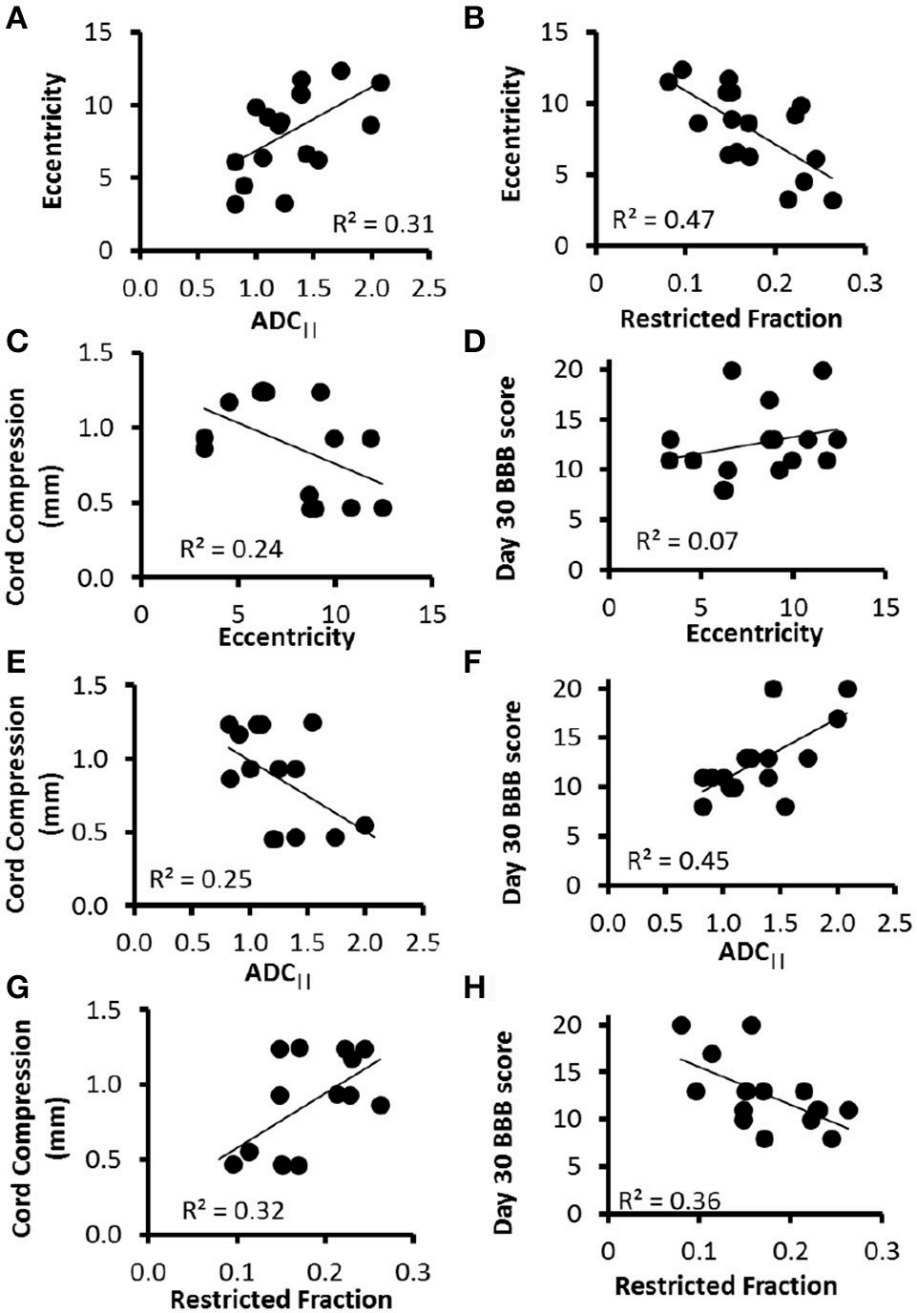
DDE-PRESS Relationship to Severity and Outcomes. Across all animals with a range of injury severities, both ADC_||_ and *f*_*R*_ measured at the injury site were moderately correlated with eccentricity **(A,B)**. The relationship with cord compression **(C,E,G)** were similar across all three parameters. The strength of the correlation with BBB score 30 days post-injury was lowest for eccentricity **(D)**, greatest for ADC_||_ **(F)**, and in between for f_*R*_ **(H)**.

In a direct comparison in the same animals, ADC_||_ and ε were significantly related (Figure 4A) to one another (*t* = 2.58, *p* = 0.021, *R*^2^ = 0.31), as were *f*_*R*_ and ε (*t* = −3.63, *p* = 0.002, *R*^2^ = 0.47) (Figure 4B). ADC_||_ (*t* = −2.01, *p* = 0.067, *R*^2^ = 0.25), *f*_*R*_ (*t* = 2.39, *p* = 0.034, *R*^2^ = 0.32), and ε (*t* = −1.95, *p* = 0.075, *R*^2^ = 0.24) had similar relationships with the compression (in mm) of the spinal cord at the moment of impact (Figures 4 C,E,G), although only *f*_*R*_ reached significance. ADC_||_ was a significant predictive marker of locomotor outcome (Figure 4F) as measured by the relationship with BBB score at 30 days post-injury (*t* = 3.39, *p* = 0.004, *R*^2^ = 0.45), and *f*_*R*_ was significant (Figure 4H) but had a lower relationship (*t* = −2.81, *p* = 0.014, *R*^2^ = 0.36). ε (*t* = 1.05, *p* = 0.31, *R*^2^ = 0.07) was not a significant predictor of locomotor outcome (Figure 4D). None of the measures of data quality were related to BBB score, including CoV (*t* = 0.05, *p* = 0.96) or FWHM (*t* = −0.05 *p* = 0.96). Moreover, neither CoV nor FWHM were significantly related to the outcome measures of ADC_||_ (CoV: *t* = −0.39, *p* = 0.70; FWHM: *t* = −0.06, *p* = 0.95) or ε (CoV: *t* = 0.18, *p* = 0.86; FWHM *t* = −1.21 *p* = 0.24).

### *In vivo* effects of single vs. double diffusion filter-probe

**Figure 5:**
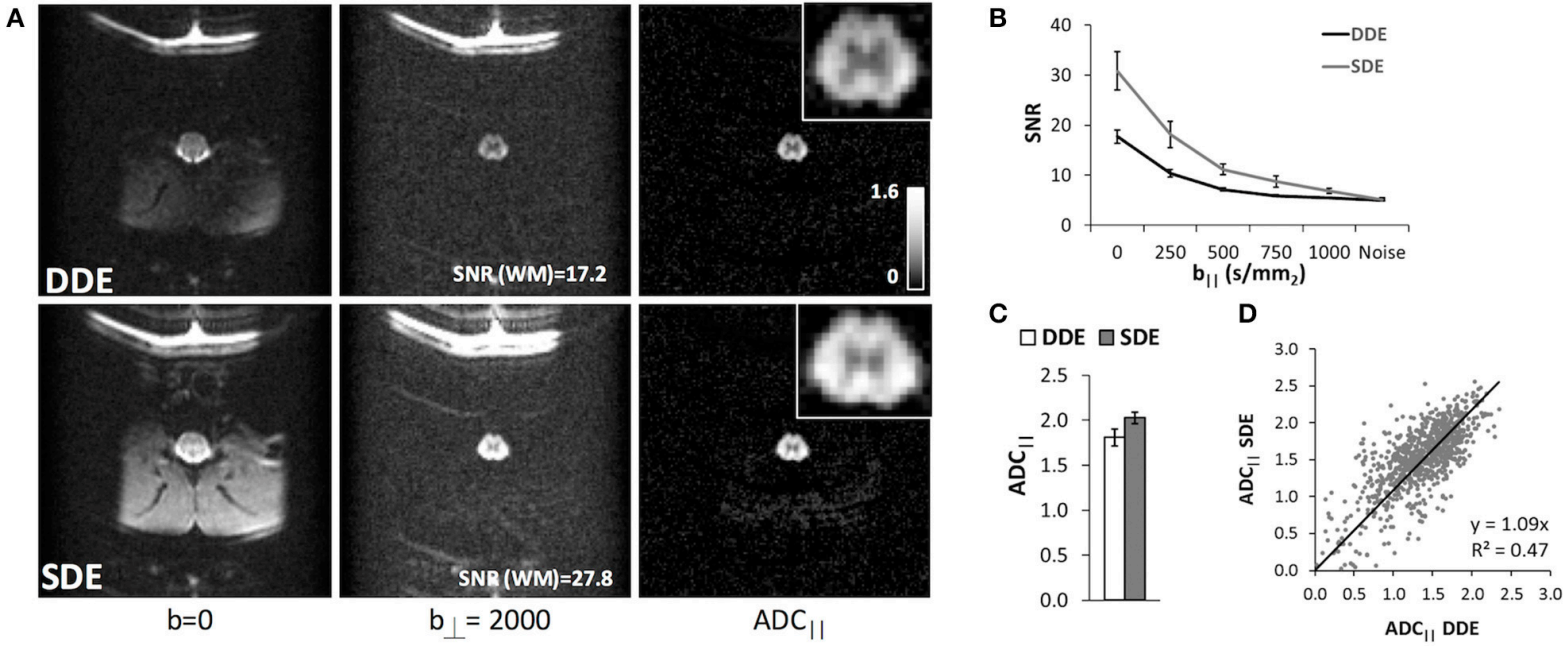
Double and Single Filter-Probe Diffusion Encoding. Representative images **(A)** from the double (top) and single (bottom) diffusion encoding variants of the DWI sequence. As expected, the SDE had improved SNR compared to the DDE acquisition. ADC_||_ maps depict primarily the spinal cord white matter along with fat. Across 3 naïve animals, the white matter signal **(B)** in the SDE remains above noise floor even at b_||_ = 1,000 s/mm^2^, whereas the DDE signal was indistinguishable from the noise above b_||_ = 500 s/mm^2^. The measured ADC_||_ of the SDE and DDE were comparable **(C)**, with a slight elevation in the SDE that could be attributable to the greater SNR. In the white matter voxels across all animals **(D)**, ADC_||_ values from the DDE and SDE were strongly correlated with a slope of 1.09.

As expected, diffusion weighted images from the SDE (Figure 5A) had a higher SNR than those with the DDE encoding due to the shorter TE for the SDE. The ADC_||_ maps from both filter-probe diffusion encoding schemes were similar and were largely devoid of non-spinal cord signals, as anticipated. Across 3 naïve animals, the mean SNR of the whole cord was 1.6 times greater in the SDE than the DDE (Figure 5B), which is consistent with the predicted SNR increase of 1.68 based solely on the reduction in TE, using a T_2_ of the spinal cord white matter of 50.2 ms. A significant increase in the white matter ADC_||_ (Figures 5C,D) was evident in the SDE compared to the DDE in a paired *T*-test (*t* = 6.04; *p* = 0.026). The difference is likely explained by the differences in T_2_ relaxation filtering. In either the SDE or DDE, no significant differences were evident from positive or negative sign combinations of diffusion gradient encoding directions.

### Application of *rFOV FP-SDE* in SCI

**Figure 6:**
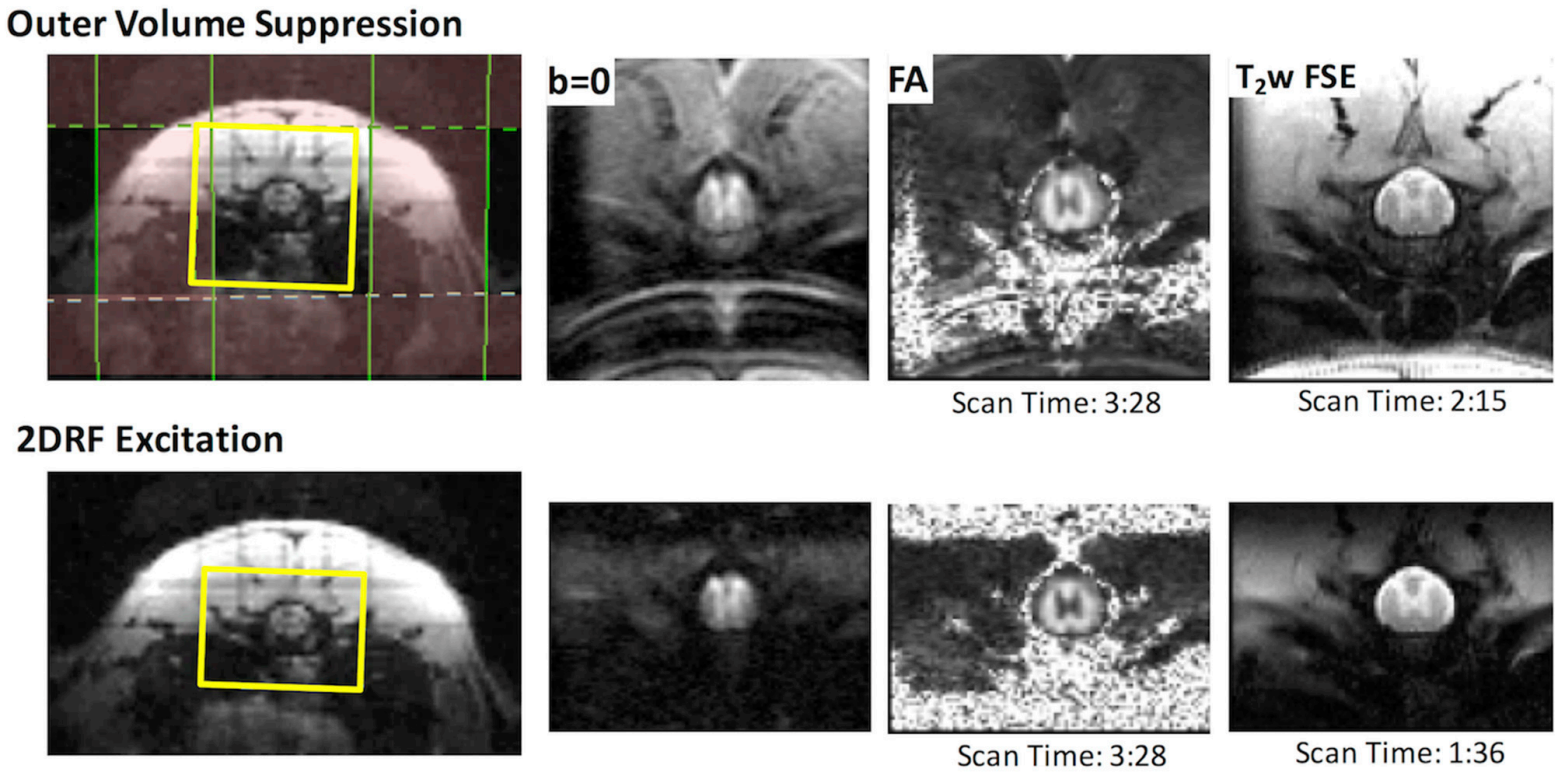
Reduced Field of View DWI and T_2_-weighted Imaging. rFOV using outer volume suppression (top) or 2DRF (bottom) provided high-quality images of the thoracic spine. Fold-over and chemical shift artifacts were evident in the OVS, which includes a separate fat-suppression module. The 2DRF with a slightly smaller FOV but an identical resolution offered similar SNR, minimal chemical shift artifacts (without separate fat suppression), and slight improvement in EPI distortion as shown by the *b* = 0 images and FA maps. In a T_2_-weighted fast spin echo, the smaller FOV allowed a reduction in acquisition time with a comparable SNR.

The rFOV-DWI using 2DRF improved image quality by reducing artifacts compared to OVS (Figure 6). The rFOV images reflected the full FOV acquired without any cropping. Notably, OVS images contained fold-over artifacts of the lipid signal even though fat suppression and spatial saturation bands were utilized. The 2DRF images contained negligible lipid signal without separate fat suppression pulses consistent with their intrinsic suppression of lipids based on the chemical shift. The measured SNR of the rFOV-DWI (47.4) was increased by approximately 12% compared to the OVS-DWI (41.5) averaged across all diffusion weighted images from a whole-cord ROI in a single animal. The rFOV had a slight improvement in image distortion, although neither the artifacts nor distortion was quantified.

**Figure 7:**
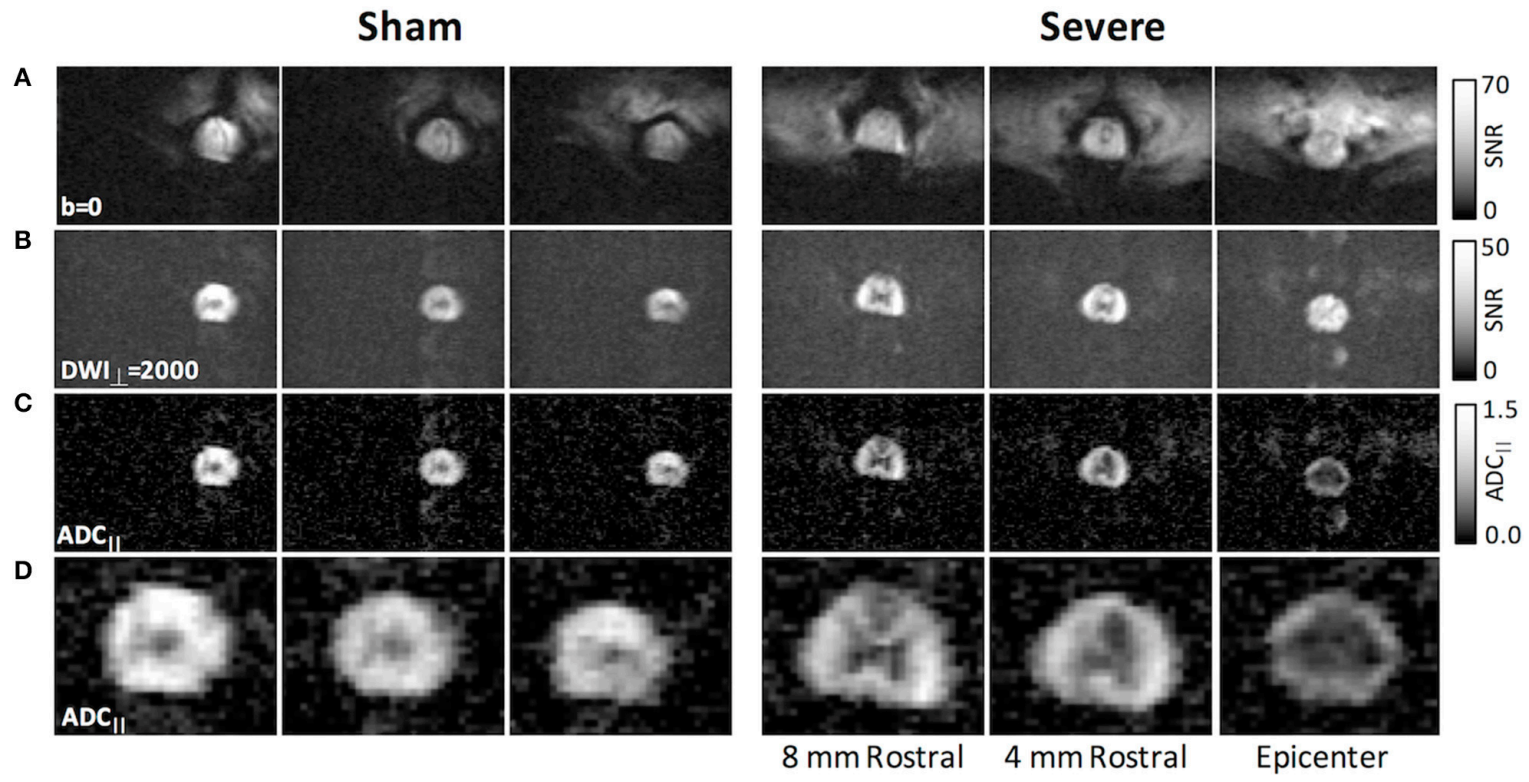
rFOV DWI in SCI with filter-probe SDE. Compared to the non-diffusion weighted images **(A)**, the perpendicular diffusion-weighted images **(B)** were free from extraneous tissue signals, although some slight EPI ghosting was evident. The filtered ADC_||_ maps **(C)** reflected primarily the intra-axonal diffusivity. In an acute severe SCI (right), the ADC_||_ maps clearly demonstrated a reduction in the central region at the epicenter and in the dorsal columns rostral to the lesion. Magnification of filtered ADC_||_ maps are shown in **(D)**.

The full pulse sequence incorporating 2DRF excitation and FP-SDE diffusion encoding was applied to rats with varying injury severities (Figure 7). The perpendicular weighted images (DWI) revealed high signal in the white matter even in an animal with a severe injury. Qualitatively, the ADC_||_ maps demonstrated a clear and pronounced effect of injury severity that was evident, with the severely-injured animal having an ADC_||_ decrease in the central region of the cord and a higher ADC_||_ rim along the periphery. It should be emphasized that the region along the periphery of the white matter would typically be obscured or confounded by the surrounding CSF. A decrease in ADC_||_ within the ascending dorsal columns is also evident in the severe injury.

**Figure 8:**
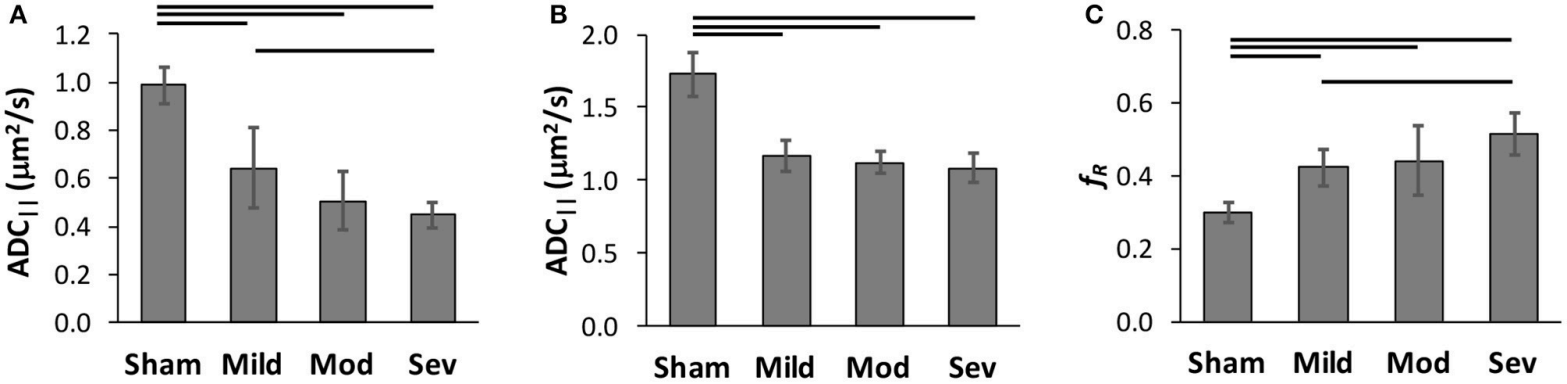
Classification of Spinal Cord Injury Severity. Region of interest analysis of FP-SDE **(A)** was compared to DDE-PRESS **(B,C)** in the same animals across a range of injury severities. ADC_||_ from imaging with a whold-cord region of interest analysis **(A)** showed an effect of injury severity. ADC_||_ obtained from single voxel PRESS **(B)** was less sensitive to severity, while *f*_*R*_ from the same voxel **(C)** better distinguished injury severity. Lines indicate significant group differences at *p* < 0.05.

A region of interest analysis was performed to quantitatively assess the DWI measures across the full cohort of animals (Figure 8). Based on the simulation results, only voxels with a SNR above 12 on the DWI images were included by masking with a whole-cord ROI in conjunction with automatic thresholding. ADC_||_ showed a significant effect of injury severity [*F*_(3, 18)_ = 10.2; *p* = 0.001] across all animals (*n* = 16) but was non-significant with the sham animals omitted [*F*_(2, 12)_ = 0.76; *p* = 0.49]. Single-voxel PRESS estimates of the same parameter were also obtained in the same animals, and all spectra had sufficient SNR to ensure robust estimates of both ADC_||_ and *f*_*R*_. (mean SNR = 69.7, range = 46.3–116.4). ADC_||_ showed a strong effect of injury severity [*F*_(3, 14)_ = 27.2; *p* < 0.0001] although it was also non-significant with the sham animals excluded [*F*_(2, 12)_ = 1.1; *p* = 0.37]. *f*_*R*_ also had a significant effect of severity [*F*_(3, 12)_ = 6.67; *p* < 0.005], but was non-significant with the sham animals included [*F*_(3, 12)_ = 2.38; *p* = 0.13].

### Human application of *rFOV FP-SDE*

**Figure 9:**
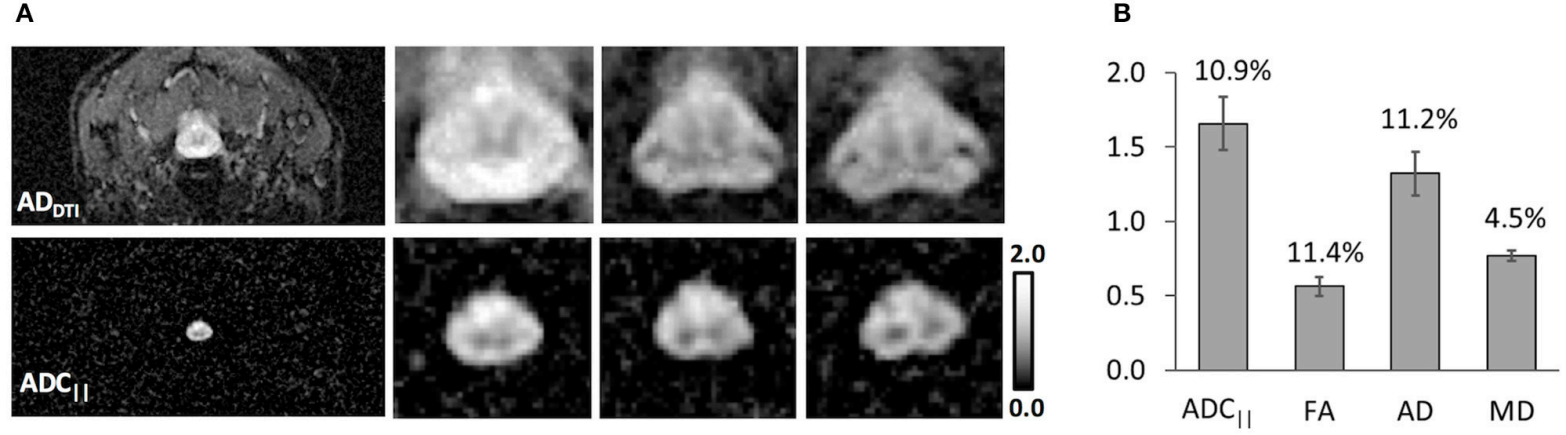
Filter-Probe diffusion weighted imaging in Human Normal Cervical Spinal Cord. The filter-probe SDE scheme was implemented on a human 3T scanner by altering the diffusion gradient orientations of the commercially-available diffusion weighted imaging sequence. Compared with AD maps derived from DTI **(A)**, FP-SDE provided comparable ADC_||_ maps but with almost complete attenuation of non-cord signals. Three of 10 slices are shown. Results from 3 healthy subjects **(B)** demonstrate similar coefficients of variation (indicated in text above bars) in the same subjects and regions of interest. ADC_||_ was significantly greater than AD from DTI. Bars indicate standard deviations.

A commercially-available diffusion weighted EPI sequence with reduced field of view (rFOV) was applied to the human cervical spinal cord using the FP-SDE diffusion encoding scheme and compared to a conventional DTI acquisition at the same resolution (Figure 9). AD_DTI_ and ADC_||_ were visually similar within the spinal cord, although as expected, ADC_||_ more clearly delineated the spinal cord due to the suppression of non-cord signals, permitting visualization of the white matter boundary without partial volume contamination by CSF. The mean filter-probe SNR in the cord white matter was 10.8 (±2.3). Across three healthy subjects averaged in all 10 slices, ADC_||_ in the spinal cord white matter (1.66 ± 0.18 μm^2^/ms) was larger than AD_DTI_ (1.32 ± 0.15 μm^2^/ms), with difference being significant in a paired *t*-test (*t* = −5.92; *p* = 0.027). In this limited sample, similar between-subject coefficients of variation were observed for ADC_||_ (10.9%) and AD_DTI_ (11.2%).

## Discussion

Following from previous work (Skinner et al., 2015, 2016), the filter-probe diffusion encoding scheme demonstrates detection of microscopic injury in the spinal cord. These studies define experimental parameters to enable improved detection of injury and translation to human spinal cord. The filter-probe diffusion encoding strategy is premised on the pathology of axonal injury manifesting as focal swellings or beading. Beading in traumatic SCI has been directly observed *in vivo* in a murine model (Williams et al., 2014), and axonal injury is consistently the pathological feature most related to functional outcome (Medana and Esiri, 2003). On the other hand, edema and cavitation are pathological features of acute and chronic spinal cord injury, respectively, yet they are not direct markers of axonal integrity. These features can confound or obscure the sensitivity of diffusion MRI to the underlying axonal injury. Edema, detected as T_2_-weighted hyperintinsities, has a strong dependence on the injury-to-imaging time in SCI (Leypold et al., 2008) is not a good predictor of SCI severity (Dalkilic et al., 2017). The filter-probe design follows from prior work using diffusion weighting as a mechanism to suppress these unwanted MR signals in addition to its more common utility as a probe of diffusivity. Importantly, a high-strength diffusion gradient perpendicular to the spinal cord attenuates fast diffusing spins, which includes free CSF, the hindered extracellular water including edema. While other methods such as fluid attenuated inversion recovery diffusion imaging (DW-FLAIR) have been used to suppress CSF (Hirsch et al., 1999), it both increases scan time and does not suppress the effects of extracellular fluid and edema.

A primary limitation of the filter-probe DWI technique is the necessity that the diffusion filter gradients are aligned perpendicular to the spinal cord. Importantly, we chose to consistently employ a diffusion filter along the left-right axis, as the spinal cord has less curvature along this axis than along the anterior-posterior axis, and although spinal cord white matter fibers are primarily aligned with the cord axis, some fiber dispersion or crossing is present. Nonetheless, considering the extent of damage following a traumatic injury, the injury is likely to be the dominant pathology affecting the diffusion measurements. The alignment of the diffusion weighted gradients relative to the underlying fibers has a cos^2^ dependence (Jespersen et al., 2010), indicating that angular deviations of up to 18 degrees will be within 10% of the true values. It was previously demonstrated in simulations that microscopic anisotropy (eccentricity) was both highly specific to beading and invariant to the underlying fiber direction. It was hypothesized that this metric would therefore retain sensitivity to axonal injury in the spinal cord and eliminate the directional dependence, albeit with an increase in acquisition time. Eccentricity had a correlation with injury severity similar to that of ADC_||_, and the two were strongly correlated to one another, which is consistent with its similar sensitivity to axonal beading (Skinner et al., 2015) in simulations. Somewhat surprisingly, eccentricity was less reliable at predicting chronic functional outcome than ADC_||_ (Skinner et al., 2015). The reasons for the discrepancy are unclear, but could be potentially related to either the underlying pathology itself or experimental and methodological considerations. The choice of diffusion weighting strength (*b*-value) and separation between the two DDE diffusion gradient pairs (mixing time;τ) may have affected the sensitivity of DDE. On the other hand, in SCI, diffusion parallel to the cord (axial diffusivity) is consistently a better marker of injury and a better predictor of outcome than FA (Kim et al., 2010; Tu et al., 2010; Shanmuganathan et al., 2017), suggesting there may be an underlying basis for a similar benefit of ADC_||_ compared to eccentricity. Although eccentricity and *f*_*R*_ were strongly correlated to one another, the two parameters reflect different features of the microstructure and may be seen as complementary. ADC_||_ and *f*_*R*_ reflect the diffusivity and restrictions, respectively, along the cord that are associated with axons, whereas eccentricity captures the shape of microstructures and may include both axonal and non-axonal features. Additional investigations into the pathophysiological basis and specificity for both DDE measures, as well as their potential applications in neurological injury and disease, are needed.

Our initial FP-DDE studies used a single-voxel encompassing the full cross section of the spinal cord at the injury site. This approach allowed rapid quantification due to its minimal and straightforward post-processing. However, as shown in Figure 7, imaging-based readouts enable visualization of the pattern of injury which may be more informative for tract-specific functional outcomes. While the two techniques are complementary, they both have distinct advantages and disadvantages that may find different applications to clinical situations. Most notably, there is always a balance between spatial resolution and SNR, and as expected (Jones and Basser, 2004; Farrell et al., 2010), the reliability of the estimated parameters depends greatly on SNR. The simulation results revealed ADC_||_ was less affected by low SNR than either eccentricity or *f*_*R*_ highlighting its potential value as a reliable parameter for imaging-based DDE acquisitions where SNR and spatial resolution are in competition with one another. Imaging, compared to a single voxel readout, obviously enables spatial information to address more detailed anatomical investigation. This study utilized a whole-cord region of interest analysis approach and semi-automated methods to reduce potential bias compared to manually-defined regions (Martin et al., 2016). Although segmentation of spared vs. injured white matter has been shown to improve separation of injury groups (Kim et al., 2010), these manual methods are subjective, labor intensive, and require a separate control group to define the normal white matter values and ranges. Currently, whole-cord regions of interest are advocated by the NIH Common Data Elements (Biering-Sorensen et al., 2015). Our decision to use a single whole-cord region of interest was based on the simplest and most straightforward approach for quantification. Automated registration and tract-specific analysis methods are useful when the cord anatomy is relatively preserved (De Leener et al., 2017), but it is unclear how well these techniques will perform with significant anatomical disruptions seen in acute human DTI of spinal cord trauma (Shanmuganathan et al., 2017). Further investigations of the different quantification approaches will be useful in combination with the optimized contrasts evaluated in this study.

Importantly, the simulations and *in vivo* results in naïve animals also demonstrated equivalence between the single and double diffusion encoding using the identical diffusion encoding schemes. The reduction in TE led to an SNR improvement of approximately 1.6. Unlike other DDE acquisitions, the SDE was only possible since the diffusion filter and probe gradients were orthogonal and assumes the fibers were coherent and aligned with the spinal cord axis. *In vivo*, a significant difference in ADC_||_ was observed between the single and double diffusion encoding, which is likely due to the effects of T_2_ relaxation since different TEs were used. One benefit of reduced TE with the SDE is an improved SNR. Likewise, a practical advantage of SDE is its compatibility with existing pulse sequences with only minor modifications to the diffusion encoding directions and amplitudes. Moreover, the filter-probe scheme utilizes a perpendicular diffusion weighted image for normalization rather than an unweighted (*b* = 0) image for reference as is done in most other diffusion analytical models. This also reduces artifacts associated with CSF signal such as flow or pulsation artifacts (Maier, 2007) and Gibbs ringing (Perrone et al., 2015).

The combined reduced field of view and FP-SDE scheme enabled high-resolution images of the spinal cord with prominent detection of injury severity on a per-subject basis. Notably, in one example ADC_||_ map from a severely-injured animal (Figure 7A), a central region of decreased ADC_||_ was evident, with a region of higher ADC_||_ along the peripheral white matter. This pathological feature is consistent with the known pathology of contusion injury (Hausmann, 2003; Kim et al., 2006; Loy et al., 2007). Compared to DTI maps of AD, the suppression of CSF in the filter-probe scheme eliminated partial volume effects along the white matter border that would otherwise be difficult to visualize (Kim et al., 2006). The DWI images, which are a surrogate measure of axonal density, did not reveal pronounced effects of injury severity at the acute timepoint. A strong relationship between ADC_||_ and injury severity was maintained in the quantitative analysis across all animals (Figure 8). Future studies explicitly modeling axonal density in concert with ADC_||_ would be useful. Furthermore, a single post-injury timepoint was assessed in this study at 48 h post-injury, which is typically longer than the initial MRI exam in acute SCI (Talbott et al., 2015; Shanmuganathan et al., 2017). Previous studies have demonstrated that diffusion indices evolve over the initial acute post-injury period, although axial diffusivity varies less than radial diffusivity within the first week (Kim et al., 2007). A more detailed examination of the post-injury window using FP-DDE would be instrumental in assessing the role of edema in these changes and translating these techniques to clinical settings where the variability in the injury-to-imaging time is expected due to the other medical complications of SCI.

Finally, in the first application to the human spinal cord, the filter-probe diffusion approach reduced partial volume effects with CSF (Figure 9). Interestingly, white matter ADC_||_ was greater than AD derived from DTI in the same subjects and regions. This insight has implications for modeling of the compartmental DWI signal models (Jelescu et al., 2015, 2016). However, it should be noted that the two measures were derived differently using either a single-axis (ADC_||_) or tensor-based (AD) estimate and should not be considered a direct investigation of the intra/extracellular diffusion properties. Importantly, through optimization of the acquisition, the variability of ADC_||_ estimates were comparable to those derived from DTI. While respiratory gating was used for all acquisitions in the rat, no gating was used for human studies despite studies demonstrating its advantages (Spuentrup et al., 2003; Mohammadi et al., 2013) evidence that CSF pulsation in the human is primarily associated with respiration (Dreha-Kulaczewski et al., 2015, 2017). However, the filter-probe approach suppresses CSF which is advantageous to reduce CSF-related artifacts (Maier, 2007). Further reliability assessments and application to patients with SCI or disease using the optimized protocol will be needed to demonstrate the reliability, applicability, and utility of the technique for research purposes or clinical diagnosis.

## Conclusions

Collectively, these acquisition advances demonstrate the sensitivity of the filter-probe diffusion weighted contrast technique to spinal cord injury. Optimizations to the acquisition and contrast mechanisms have been refined and examined for human translation, culminating in implementation of the FP-SDE scheme on clinical scanner systems without significant modifications. The strong association between quantified diffusivity values and functional outcomes in a rat model of SCI shows high sensitivity to acute injury with the potential to be useful in clinical evaluation of SCI. Future applications to patients with injury and disease of the spinal cord will refine the clinical applicability filter-probe DWI to diagnosis and prognosis.

## Ethics statement

This study was carried out and approved by the Institutional Review Board of the Medical College of Wisconsin. All subjects gave written informed consent in accordance with the Declaration of Helsinki. This study was carried out and approved by the Institutional Animal Care and Use Committee of the Medical College of Wisconsin and the Clement J Zablocki VA Medical Center.

## Author contributions

MDB, NS, and LM: Acquired Data. MDB, NS: Conducted Analysis. All Authors: Edited and Approved Manuscript.

### Conflict of interest statement

The authors declare that the research was conducted in the absence of any commercial or financial relationships that could be construed as a potential conflict of interest.
